# Insight into the Structure, Dynamics and the Unfolding Property of Amylosucrases: Implications of Rational Engineering on Thermostability

**DOI:** 10.1371/journal.pone.0040441

**Published:** 2012-07-06

**Authors:** Ming Liu, Shuang Wang, Tingguang Sun, Jiguo Su, Yuanxing Zhang, Junjie Yue, Zhiwei Sun

**Affiliations:** 1 Beijing Institute of Biotechnology, Beijing, China; 2 State Key Laboratory of Bioreactor Engineering, East China University of Science and Technology, Shanghai, China; 3 Genor Biopharma Co., Ltd, Shanghai, China; 4 Department of Biological and Chemical Engineering, Guangxi University of Technology, Liuzhou, China; 5 College of Science, Yanshan University, Qinhuangdao, China; National Institute for Medical Research, Medical Research Council, London, United Kingdom

## Abstract

Amylosucrase (AS) is a kind of glucosyltransferases (E.C. 2.4.1.4) belonging to the Glycoside Hydrolase (GH) Family 13. In the presence of an activator polymer, in vitro, AS is able to catalyze the synthesis of an amylose-like polysaccharide composed of only α-1,4-linkages using sucrose as the only energy source. Unlike AS, other enzymes responsible for the synthesis of such amylose-like polymers require the addition of expensive nucleotide-activated sugars. These properties make AS an interesting enzyme for industrial applications. In this work, the structures and topology of the two AS were thoroughly investigated for the sake of explaining the reason why *Deinococcus geothermalis* amylosucrase (DgAS) is more stable than *Neisseria polysaccharea* amylosucrase (NpAS). Based on our results, there are two main factors that contribute to the superior thermostability of DgAS. On the one hand, DgAS holds some good structural features that may make positive contributions to the thermostability. On the other hand, the contacts among residues of DgAS are thought to be topologically more compact than those of NpAS. Furthermore, the dynamics and unfolding properties of the two AS were also explored by the gauss network model (GNM) and the anisotropic network model (ANM). According to the results of GNM and ANM, we have found that the two AS could exhibit a shear-like motion, which is probably associated with their functions. What is more, with the discovery of the unfolding pathway of the two AS, we can focus on the weak regions, and hence designing more appropriate mutations for the sake of thermostability engineering. Taking the results on structure, dynamics and unfolding properties of the two AS into consideration, we have predicted some novel mutants whose thermostability is possibly elevated, and hopefully these discoveries can be used as guides for our future work on rational design.

## Introduction

Amylosucrase (AS) is a kind of glucosyltransferase (E.C. 2.4.1.4) belonging to the Glycoside Hydrolase (GH) Family 13 according to the Carbohydrate-Active EnZymes classification [1], [2], [3]. The first AS was identified in *Neisseria perflava* as early as 1946 [4]. Three to four decades later, MacKenzie *et al*. [5] identified intracellular AS in six other *Neisseriae* species, and later an extracellular *Neisseria polysaccharea* AS was discovered [6]. Until recently AS has only been found in bacteria from the genus *of Neisseria* [7], *Deinococcus* [8], [9] and *Alteromonas* [10]. However, *Neisseria polysaccharea* amylosucrase (NpAS) is the only AS for which several structures, alone or in complex with sucrose substrate or products are available to date [11], [12], [13], [14]. According to crystal structures, AS possesses the characteristic (β/α)_8_-barrel catalytic A-domain, a B-domain between β3 and α3, and a C-terminal domain consisting of a sandwich of two Greek key motifs. In addition to these common structural features of GH13, AS also possesses two unique domains: an α-helical N-terminal domain and a B′-domain between β7 and α7 in the catalytic core, which has been suggested to be involved in the polymerase activity of this enzyme. The B and B′-domains contribute largely to the formation of the active site pocket [7].

Unlike many other enzymes of the GH13, which mainly degrade polyglucan, AS is a very exceptional one since its main role is to catalyze the produce of the insoluble polysaccharides. In the presence of an activator polymer i.e. glycogen, in vitro, AS is able to catalyze the synthesis of an amylose-like polysaccharide composed of only α-1,4-linkages using sucrose as the only energy source [15]. In the absence of glycogen, the reaction pathways however become much more complicated, including polymer synthesis, hydrolysis of sucrose, synthesis of smaller maltosaccharides, and synthesis of sucrose isoforms [7]. Hydrolysis, however, is always a minor side reaction under each circumstance. The function of AS *in vivo* is undoubtedly the extension of glycogen-like oligosaccharides, which is clearly demonstrated by the formidable increase in *k*cat observed when glycogen is present [16]. In contrast to AS, other enzymes responsible for the synthesis of such amylose-like polymers require the addition of expensive nucleotide-activated sugars such as ADP- or UDP-glucose [17]. Amylosucrase can also be used to modify the structure of polysaccharides such as glycogen by the addition of α-1,4-linked glucosyl units [18]. These properties make AS a kind of interesting and promising enzyme for industrial applications. 

Among the AS discovered so far, NpAS is the one which is studied most. Although of great potential for industrial applications, NpAS suffers from a low catalytic efficiency on sucrose alone (*k*_cat_ = 1·s^−1^) and a weak thermostability (t_1/2_[50°C] = 3 min), limiting its industrial development [9]. Directed evolution has been attempted to improve catalytic efficiency and thermostability of NpAS [19]. Searching for more thermostable and efficient enzymes in the natural diversity is another alternative that has motivated the biochemical characterization of the AS from *Deinococcus geothermalis* (DgAS) [9], and *Deinococcus radiodurans* (DrAS) [8]. DrAS possesses similar stability and activity properties with NpAS. With a specific activity of 44 U.mg^−1^ at the optimal temperature of 50°C, the recombinant DgAS is the most thermostable AS characterized to date [9].

For the sake of engineering a protein for higher thermostability, many rational approaches have been developed. According to whether the tertiary structure information will be used, the rational design can be roughly divided into two classes. One is the sequence-based method, and the other is the structure-based method. The sequence-based method requires only the information of the target and some templates sequences, therefore, is quite useful when the structure of the target protein is not available. As one of the most popular methods of the sequence-based class, sequence alignment between the mesophilic and the thermophilic proteins can provide some useful information for engineering [20]. The Local Structure Entropy (LSE) is a good measurement for the intrinsic thermostability of a protein [21]. Joint using of the two methods has been proved as an effective way to engineer the thermostability of proteins [22]. Despite of the advantages of these sequence-based methods, some protein scientists complain that the engineered proteins frequently end up with inactivation or even misfolding, not mention to obtain higher stability. This is, however, not surprising to us, since using the sequence based method alone is often not good enough for a successful design. Using this kind of method alone, obviously, may affect the subtract binding interface or the interaction network of some specific regions, since these methods consider the sequence information only. The actual world of protein, however, is three-dimensional. In order to improve the design accuracy, sequence-based methods are frequently coupled with structure-based ones. Structure-based class contains methods that utilize the tertiary structure information of the target or template proteins, such as molecular dynamics (MD) simulation, coarse-grained models, molecular docking and modeling. In many cases, using structure based alone is good enough for thermostability engineering already.

For thermostability elevation, understanding of the structural and dynamics differences between the mesophilic and the thermophilic proteins are of great importance. MD simulation is a powerful method and especially useful when some specific residues play a role in protein function and activity [23], [24], [25]. These simulations are however computationally costly because energy calculations need to be repeated at each femtosecond-scale (10^−15^ seconds) step and reaching biologically meaningful time scales, i.e. >10^6^ seconds, can be challenging for real proteins. In comparison with MD simulation, elastic network models (ENM) that use coarse-graining at the amino acid-level with normal mode analysis (NMA) proved powerful in deciphering biologically meaningful motions [26], [27], [28]. The Gaussian network model (GNM) is the simplest form of ENM. This is an elastic network (EN) model introduced at the residue level [29], [30], inspired by the full atomic NMA with a uniform harmonic potential [31]. Despite its simplicity, the GNM and its extension, the anisotropic network model (ANM) [32], or similar coarse-grained EN models combined with NMA [33], [34], [35], have been widely uses as useful tools for elucidating the functional motions [24], [36], the kinetically hot residues [36], [37], protein assemblies and many other dynamics properties which are hard or even impossible to study by the classical MD simulations [38], [39], [40], [41]. Besides, we have reported a GNM based method for protein unfolding pathway prediction, with which one can find out the kinetically ‘“weak” regions of the target protein with reasonable accuracy [42], [43].

Two main problems we want to solve in the work are what constitute the superior thermostability of DgAS and how we can utilize the features of one AS to engineer another? To figure out the two problems, the GNM and ANM methods are applied to gain insight into the dynamics and unfolding properties of NpAS and DgAS. Furthermore, the structures of these two proteins are carefully and thoroughly investigated, for the purpose of finding out the structural basis lying behind the differences in thermostability of the two proteins. Hopefully the results revealed in the current work can be used as guides for practical developments on the AS in the further.

## Materials and Methods

### Input Structures

Searching Protein Data Bank (PDB, http://www.rcsb.org) [44] with the key word of “amylosucrase”, totally thirteen AS structures could be retrieved by the moment we began this work (around Jan, 2012). Among the thirteen structures, only the newly resolved two (3UCQ and 3UER [45]) are of DgAS, and the other eleven PDB files belongs to NpAS.

Among the eleven structures of NpAS deposited in the PDB, 1G5A is of the highest resolution (1.4 Å), and the other ten's resolutions range from 1.66 to 2.2 Å. All of these PDB files, except 3UEQ contain 628 (1–628) residues. By scrutinizing the PDB file of 3UEQ, the first four residues are merely the residual expression tag. 3UEQ, therefore, is also of the same length of the other ten structures of NpAS. In this work, 1G5A [12] was selected as the input of the following analyses since its high resolution.

The tertiary structure of DgAS was just published on the PDB by the end of 2011. To date, only two structures are deposited in the PDB. Both 3UCQ and 3UER contain 651 residues, form −4 to 646, and the first five residues in the two structures are the residual expression tag. 3UCQ was used in this work as the input, and the residual expression tag was removed to construct a more reasonable model. Besides, there are four missing residues, i.e. _647_EAPA_650_, at the tail of the C-domain. These missing residues were added to complete the DgAS structure with MODELER [46], [47], [48], [49] and the conformation of these residues were minimized by NAMD [50] with the other residues restrained in their native positions. The minimization protocol used here is similar with that employed in our previous studies on other proteins [23], [24], [25]. The final input structure of DgAS contains 650 residues, and the residue index ranges form 1 to 650.

### GNM and ANM

The GNM was first proposed by Bahar et al. [29] within such a minimalist mindset to explore the role and contribution of purely topological constraints, defined by the 3D structure of protein. The GNM describes a three-dimensional structure of protein as an elastic network of C_α_ atoms connected by harmonic springs within a certain cutoff distance. The cutoff distance is usually taken as 7.0 Å, based on the radius of the first coordination shell around residues observed in PDB structures [51], [52].The force constant is identical for all springs. Considering all contacting residues, the internal Hamiltonian of the system can be defined as [53]:
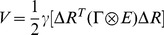
(1)where 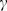 is the force constant of the harmonic spring connecting each node; 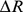 represents the 3N-dimensional column vector for fluctuations, and 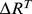 is its transpose; 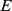
is the third-order identity matrix; 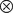 represents the operation of direct product; and 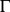 is the 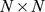
symmetric Kirchhoff matrix in which the elements are defined as [30], [53]:
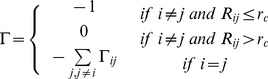
(2)where 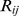 is the distance between the *i*th and *j*th C_α_ atoms and 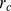 is the cutoff distance (7.0 Å is used in the current work). The mean-square fluctuations of each C_α_ atom and the cross-correlation fluctuations between different atoms are in proportion to the diagonal and off-diagonal elements of the pseudo inverse of the Kirchhoff matrix. The pseudo inverse of the Kirchhoff matrix can be decomposed as:
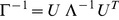
(3)where 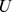 is an orthogonal matrix whose columns 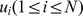 are constituted by the eigenvectors of 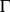, and 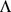 is a diagonal matrix whose diagonal consists of all 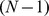 non-zero eigenvalues 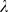 of 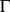. The cross-correlation fluctuations between the *i*th and *j*th residues are given by:
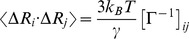
(4)where 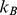 is the Boltzmann constant, 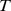 is the absolute temperature, and the meanings of 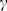 and 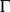 are the same as in Eq. 1. When *i* = *j*, Eq. (4) stands for the mean-square fluctuations of the *i*th residue. The Debye-Waller or B-factor, which is correlated to the mean-square fluctuation, can be calculated with the following expression:
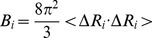
(5)Sometimes, one also wants to decompose the fluctuations onto individual modes, and the mean-square fluctuation of the *i*th residue associating with the *k*th mode is given by:
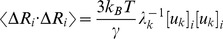
(6)In the GNM, the cross correlation is given by:
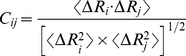
(7)GNM is more accurate when evaluating the deformation magnitudes, or the distribution of motions of individual residues [54]. In contrast, ANM is the only possible, however less realistic, model when it comes to assessing the directions or mechanisms of motions [54]. Then ANM is introduced here to assess the slow motion modes of the two AS. In ANM, the motion mode of a protein is determined by a Hessian matrix 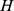:
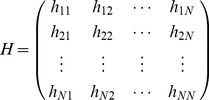
(8)where each 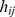 is a 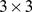 sub-matrix, which is defined by:
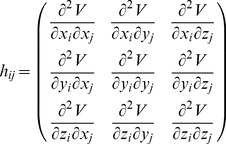
(9)when 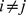, the elements of 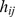 can be calculated with the following analytical expression:
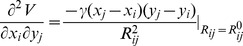
(10)when 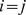, the analytic expression for the elements of 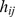 is
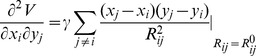
(11)The definitions of 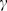 and 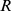 are the same as in Eq. (1). *x, y* and *z* represent the coordinates of atoms.

### Iterative GNM

The traditional GNM can only explore the dynamics of the target protein with a specific conformation. By breaking each “weak” contact between nodes step by step, we have shown the unfolding processes of proteins can be predicted by the iterative GNM, with a reasonable accuracy [42], [43].

In this work, the iterative GNM is employed to predict the unfolding behavior of the two AS. When applying the iterative GNM, the interactions between residues are divided into the covalent and the non-covalent ones. In contrast to the traditional GNM, the interactions between the covalent and the non-covalent bonded ones are treated differently. The spring constants between all pairs of non bonded residues within the cutoff distance are treated equally, i.e. a single force constant 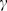 is employed. The strengths of the interactions between all covalently bonded pairs long the chain backbone are defined by 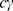, where 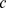 is coefficient and can be determined by fitting predicted fluctuations against the crystallographic B-factors [42]. Considering all contacting residues, the internal Hamiltonian of the system can be also defined by Eq. (1), and the newly defined Kirchhoff matrix for the iterative GNM is given by:
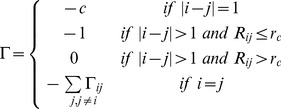
(12)where 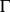, 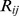 and 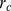 are defined as the same of those in the traditional GNM.

The mean-square fluctuation in the distance vector 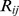 between the residues *i* and *j* can be calculated by with [55]:
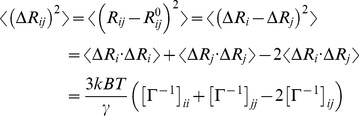
(13)where 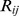 and 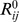 stand for the instantaneous and equilibrium separation vectors between residues *i* and *j*, respectively.

As the temperature of the system is gradually increased, the native contacts among residues are expected to break in a fluctuation-dependent manner [42]. The philosophy held by this method, therefore, is that the weakest interaction will be broken first. In order to take the stochastic nature of thermal denaturation into consideration, one can incorporate some noise into the calculations during the real simulation processes [42]. In this work, the native contact to be removed in each step was randomly selected from contacts with the first three largest fluctuations, and the calculations were performed 100 times for each AS to get more reasonable results.

The fluctuations in the distance between all residues are calculated based on Eq.(13). The nonlinear elasticity during protein unfolding is considered through iterative normal mode calculations, thus we can mimic the protein unfolding process by using the following procedure:

- The mean-square fluctuations of the distance in all residue pairs are calculated based on the native structure topology with Eq. (13).
- The contact in the residue pair with the largest distance fluctuation is broken. Then, a new matrix 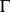 is obtained, which represents a new topology during protein unfolding.
- The mean-square fluctuations of the distance in all residue pairs are recalculated based on the new matrix 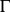 using Eq. (13).
- The above two steps are repeated until all the non-covalent contacts are broken.
- All the topologies of different conformation during protein unfolding are obtained and the unfolding pathway can be derived from the above obtained data.

### Free Energy Estimation

In order to evaluate the thermodynamics stability of the wild type (WT) AS and mutants, the folding free energy changes are estimated by the FoldX program [56], [57], [58]. FoldX uses a full atomic description of the structure of the proteins. The different energy terms taken into account in FoldX have been weighted using empirical data obtained from protein engineering experiments. The FoldX energy function includes terms that have been found to be important for protein stability. The folding free energy (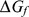) of a protein is calculated using the following equation [56], [57], [58]:
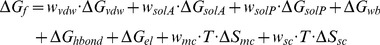
(14)where 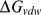 is the sum of the van der Waals (VDW) contributions of all atoms with respect to the same interactions with the solvent. 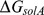 and 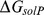 are the differences in solvation energy for apolar and polar groups respectively when these change from the unfolded to the folded state. 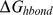 is the free energy difference between the formation of an intra-molecular hydrogen bond compared to inter-molecular hydrogen-bond formation (with solvent). 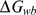 is the extra stabilizing free energy provided by a water molecule making more than one hydrogen bond to the protein (water bridges) that cannot be taken into account with non-explicit solvent approximations [59]. 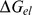 is the electrostatic contribution of charged groups, including the helix dipole. 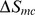 is the entropy cost of fixing the backbone in the folded state; this term is dependent on the intrinsic tendency of a particular amino acid to adopt certain dihedral angles [60]. Finally 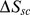 is the entropic cost of fixing a side chain in a particular conformation [61]. The energy values of 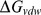, 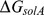, 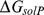 and 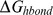 attributed to each atom type have been derived from a set of experimental data, and 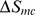 and 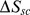 have been taken from theoretical estimates. The terms 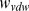, 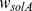, 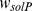, 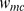 and 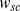 correspond to the weighting factors applied to the raw energy terms. They are all 1, except for the van der Waals' contribution which is 0.33.

The predictive power of FoldX forcefield has been tested on a very large set of point mutants (1088 mutants) spanning most of the structural environments found in proteins. In a recent comparison study on the accuracy and applicability of several energy functions, the overall performance of FoldX beat many other functions. The performance of FoldX on predictive accuracy is quite impressive, whose accuracy is as high as 70% when the crystal structure is available [62].

## Results

### I. Detailed Comparison between the Structures of NpAS and DgAS

As has been stated above, the PDB files of NpAS and DgAS used in this study contains 628 and 650 residues, receptively. Since there are many insertions and deletions in the two AS comparing with each other, the normal sequence alignment method available in most software can not guarantee to give a completely correct alignment. To overcome this problem, we firstly superimposed the structure of DgAS onto that of NpAS with the Iterative Magic Fit (IMF) method provided in the Swiss-PDBViewer [63], then the sequence alignment was adjusted on the basis of the structure superimposition. The sequence identity of two AS is 40.0%, and the root mean square deviation (RMSD) of C_α_ atoms between the two input structures is 1.14 Å for the 563 common positions. The largest difference between our sequence alignment and the one provided by Guérin *et al.* [45] is that there is a 8-residues insertion, i.e. _16_TPEQRAGI_23_, in the N-domain of NpAS. Besides, one thing should be mentioned here is that the alignment for the residues at the very beginning of NpAS and DgAS are not associated well with the structure information, since the local structures can not be superimposed well.

A verity of differences in structures have been identified between thermophilic and mesophilic proteins sharing the similar functions, which are believed to be, at least partially, responsible for the differences in their thermostability. Here the thermostability is synonymous with the heat resistance of a protein, and commonly measured by the half life at a specific temperature or the half inactivation temperature of the protein. A thermostable protein is generally believed to have larger hydrophilic solvent accessible surface areas (SASA), more H-bonds and ion-pairs, higher proline and lower non-positive-ϕ glycine proportions. For the sake of gaining insight into the differences in structure between the two AS, the factors mentioned above were calculated under same conditions. The results are listed in Table 1 and will be discussed in great details in the following paragraphs.

**Table 1:** Comparison between the structures of NpAS and DgAS.

	NpAS	DgAS
SASA total (Å^2^)	24027	24664
SASA hydrophobic (Å^2^)	3815 (15.9%)	4914 (20.0%)
SASA hydrophilic (Å^2^)	13911 (57.9%)	14283 (57.9%)
No. H-bonds^a^	245 (150/95)	255 (180/75)
No. Salt-bridges^b^	41	47
No. Glycine residues	41 (6.5%)	49 (7.5%)
No. Proline residues	30 (4.8%)	36 (5.5%)

a The distance and the angle cutoffs used for the calculation of H-bonds are 3.0 Å and 150°, respectively. The first number of the content in the bracket is the count of backbone-backbone H-bonds, the second one gives the count for the sidechain-sidechain/backbone H-bonds.

b The distance cutoff used for the calculation of salt bridges is 3.5 Å.

#### Differences in SASA, H-bonds and Salt-bridges Associate with the Discrepancy in Thermostability

Since exposed hydrophobic residues have bad solvation energy and are more prone to aggregate than polar residues, in general, a thermostable protein should have less exposed hydrophobic residues than its mesophilic counterpart. In this work, however, it is found that the SASA for the hydrophobic residues of DgAS is much larger than that of the NpAS (Table 1). This is not surprising because that the DgAS forms a homologous dimer in solvent, and the SASA of the hydrophobic residues around the dimer interface is 814 Å^2^. If we exclude this part from calculation, the SASA of the hydrophobic residues of DgAS is in the same level of that of the NpAS. This result, thereby, suggests that the solvation energy of DgAS is not necessarily worse than that of the NpAS. In order to figure out this problem, the unfolding free energies of the two AS were estimated with the FoldX program. The absolute values of the free energies are meaningless since the FoldX forcefield is not scaled to fit the real free energy, but the relative difference between calculations is meaningful. According to the calculation (Table 2), the solvation energy of DgAS is a little bit lower than that of the NpAS. Consequently, these results implicate that DgAS is at least as soluble as NpAS.

**Table 2:** Estimated folding free energies (kcal·mol^−1^) for NpAS and DgAS.

	ΔG_total_	ΔG_HB_ a	ΔG_HS_ b	ΔG_vdw_	ΔG_elec_ c	ΔG_sol_	ΔG_S_ d	ΔG_Other_ e
NpAS	−132.19	−455.10	−252.27	−815.74	−43.92	−2.53	1398.95	−11.06
DgAS	−142.63	−481.27	−225.91	−833.33	−55.44	−5.13	1421.98	−18.74

a H-bond energy for the backbone-backbone type.

b H-bond energy for the sidechain-backbone and sidechain-backbone type.

c Electrostatics energy contributions of the charged pairs.

d Energy contribution by conformational entropy at room temperature.

e Helix dipole (mainly) and others.

From Table 1, one can see that the total number of H-bonds of the DgAS is just a little bit more than that of the NpAS. Besides, we also notice that the total H-bond energies of the two AS are of the same level (Table 2), suggesting that the H-bonds energy contributing similarly to the total folding free energy of the two AS. When decomposing the total H-bonds into the backbone-backbone and the sidechain-sidechain/backbone types, it is found that the backbone-backbone H-bonds of DgAS are much more than that of the NpAS (Table 1). Nevertheless, the H-bonds formed between sidechain and sidechain/backbone for NpAS are more than those for the DgAS. These results are consistent well with the energy calculations for H-bonds in Table 2. In general, the H-bonds formed between backbone oxygen and nitrogen atoms are of good geological conditions owing to the tight restraint, therefore are possibly more stable than those formed between sidechain and sidechain/backbone atoms. Additionally, the sidechain H-bond energy might be intrinsically over estimated since sidechains were refined to more energetically favored conformations before calculations. In contrast, the atoms of backbone are restrained to stay around their native positions. Based on these results, it is suggested that the difference in H-bonds partly account for the different thermostability of the two AS.

As is well known, the polar residues are prone to be distributed on the surface of the protein because burying a polar residue in the interior of a protein will cost huge solvation energy which generally can not be compensated by other forces, such as the H-bonds formed among the buried polar residue and other residues [64]. Here we define the Asp, Asn, Glu, Gln, Lys, Arg and His as the hydrophilic residues, and the buried hydrophilic residues for NpAS and DgAS are 45 and 46, respectively. Theoretically, the maximum number of H-bonds for the buried hydrophilic residues of NpAS and DgAS are 158 and 154, yet the actual number for the two AS are just 45 and 48, respectively. Anyway, the ratio of actual H-bonds to maximum H-bonds for the buried hydrophilic residues of DgAS is higher than that of the NpAS. This is may be another factor contributing the higher thermostability of DgAS. Furthermore, the salt bridges formed in NpAS are also less than those formed in DgAS. This is not only in accordance with the common sense on the differences between the thermophilic and mesophilic proteins, but also fit well with the corresponding energy calculations shown by Table 2. Combining these results together, the differences in the intra hydrogen bonds and salt-bridges between the two AS can partially explain their difference in thermostability.

#### Some Glycine Residues are Important for the Thermostability

**Figure 1:**
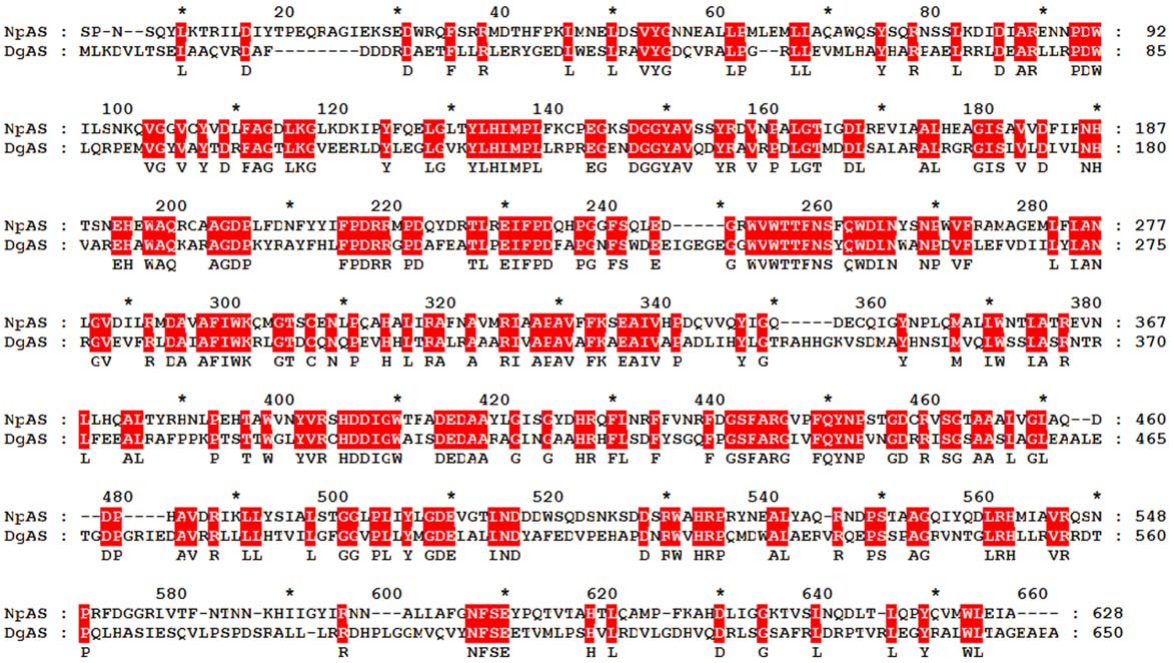
The structure based sequence alignment for NpAS and DgAS. The conserved residues are highlighted by red background.

**Figure 2:**
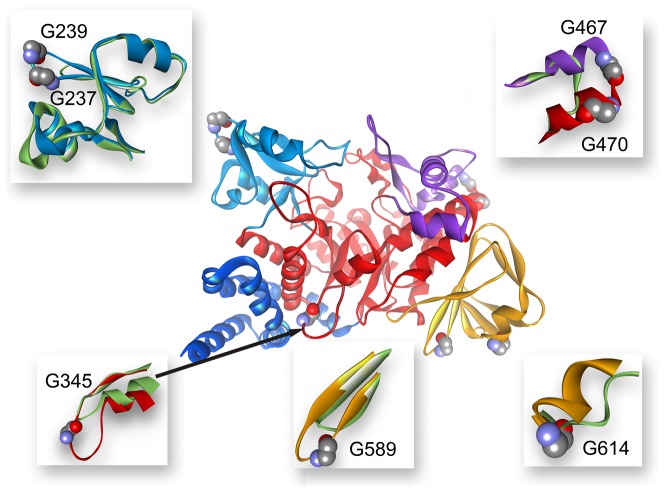
The seven additional glycine residues of DgAS. The additional glycine residues are represented by the CPK model. The N, A, B, B′ and C-domains of DgAS are colored by deep blue, red, sky blue, purple and orange, respectively. In the five inset figures, the local structures of DgAS are superimposed onto the corresponding parts of NpAS and the local structure of NpAS are colored by grass blue for clarity.

Glycine, with the smallest size, is the most flexible one among the 20 naturally occurring amino acids. It usually occurs at turns or loops of proteins, and can access much larger conformational spaces than any others. Owing to its small size, glycine is prone to facilitate the motions of the local structures around them and therefore, increase the conformational entropy of any state. Since glycine residue is the only one whose backbone can adopt 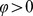 without any steric conflict, it should, as far as possible, occur at the right half of the Ramachandran plot. As for the glycine residues with negative 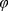, they should be replaced by other residues in order to elevate the thermostability if only the space around it is large enough. For the sake of convenience, we nominate the glycine residues with positive 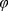 as 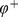 glycine residues, and the ones with negative 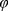 as 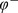 glycine residues. Surprisingly, the DgAS possesses more glycine residues than the NpAS does (Table 1). What's more, there are 21 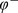 glycine residues in DgAS, which are also more than those in NpAS. Based on the structure information and the sequence alignment (Figure 1), there are 7 additional glycine residues locating at the insertions of DgAS. Among the 7 additional glycine residues of DgAS, G239, G345 and G470 are 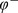 glycine residues, and the other four, i.e. G237, G467, G589 and G614 belong to 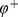 glycine residues. The positions of these glycine residues are depicted in Figure 2.

G237 and G239 locate at the turn between two β-strands of the B-domain. Substitute G237 with any other naturally-occurring residues may cause steric hindrance since it belongs to 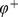. Despite of locating at the 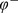 dihedral space, G239 is also important for the maintenance of the local structure, since its backbone dihedrals locate at the disallowed region for any other residue. Indeed, the energy calculation just validates our assumptions (Table 3). G467 and G470 are situated at the short loop connecting the α7 helix and the short α helix of B′-domain. G467 belongs to 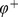, and thus in general can not be substituted by any other residues. As to G470, it can be substituted by larger residue, such as alanine, without obvious steric clash according to energy calculation (Table 3). G614 and G589 are located at a β-turn and a short loop of the C-domain, respectively. These two glycine residues are likely indispensable for the local folding and probably can not be changed to other residues without affecting the local folding. According to the energy calculation, substituting G614 or G589 with alanine residue may largely reduce the thermostability of DgAS. Finally, although the dihedral of the backbone of G345 is within the favorite region of non-glycine residues, changing it to larger residues may cause large clashes thus affecting the thermostability of DgAS (Table 3).

**Table 3:** Virtual ala-scan for the seven additional glycine residues of DgAS.

Position	Phi/Psi (degree)	Classification	ΔΔG(kcal·mol^−1^)
G237	71.15/−177.52	*ϕ*+	2.66
G239	−92.62/−136.91	*ϕ*−	1.33
G345	−69.60/171.83	*ϕ*−	1.74
G467	82.59/−12.10	*ϕ*+	3.56
G470	−70.78/−41.57	*ϕ*−	−0.60
G589	122.12/142.00	*ϕ*+	2.21
G614	86.25/−165.42	*ϕ*+	3.65

In conclusion, most of the additional glycine residues of DgAS are indispensable and can not be simply substituted by other residues without affecting the local folding and the thermostability. According to the results of virtual ala-scan on all glycine residues of DgAS and NpAS, only a very few of glycine residues are thought to be replaceable (Table S1), suggesting glycine residues are important for the overall folding and thermostability of the two proteins. Consequently, it seems that the glycine proportion of a protein is not necessary a criterion for the discrimination between the stable and the less stable proteins.

#### Proline Residues are Critical for the Thermostability of the Two AS

Proline is the most special one in structure among the 20 naturally occurring amino acids. When being a building block of a peptide or protein, proline residue is apparently the most rigid residue whose 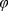 is usually restrained around 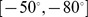, and 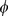 is restrained within 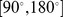 or 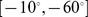. In general, proline residue is always located at the connecting region between two regular secondary structures because its backbone nitrogen atom can not work as a hydrogen bond donor. Because of the rigidity of proline residue, it can tightly hold the local structure together, and therefore the conformational entropy both of the folded and the unfolded state are highly decreased. Since the unfolded state is an ensemble of numerous non-native conformations, the entropy lost in the unfolded state is much more than those in the folded state, thus substituting a residue in a proper position with the a proline residue can theoretically enhance the thermostability of a protein.

As is shown in the last row of Table 1, the proline proportion of DgAS is higher than that of NpAS. According to our experience on calculation, substituting any residue with proline at a proper position can lower the folding free energy by 0.5 to 2.5 kcal·mol^−1^, which are consistent with Némethy and Matthews's results [65], [66]. DgAS has six more proline residues than NpAS. These additional proline residues, therefore, may lower the unfolding free energy of DgAS by 3.0 to 15.0 kcal·mol^−1^ according to our experience on calculations. Although the specific values for the changes in free energy may be not accurate, we believe these additional proline residues make positive contribution to the thermostability of DgAS. In order to validate our estimations, all proline residues of DgAS and NpAS were mutated to alanine residues, and the differences in free energy of unfolding were calculated with FoldX. The results are listed in the Table S2. According to the calculations, one can tell that proline residues indeed play critical roles in the stabilization of the two AS. All of the Pro-Ala substitutions for the NpAS are destabilized from 0.8 to 3.3 kcal·mol^−1^, and those for the DgAS are from 0.5 to 3.0 kcal·mol^−1^. To explore the this problem further, the correlation between thermostability and proline proportion for the two AS are discussed in great detail in following paragraphs.

According to the structure-based sequence alignment (Figure 1) and the structures themselves, one can figure out the proline distributions for the two AS among each domain. The detailed results are listed in Table 4. As shown in Table 4, proline residues distribute among domains with a non-uniform manner, no matter in NpAS or in DgAS. In NpAS, the B-domain holds 5 proline residues, which takes up 6.6% of the domain. Since the high composition of proline residue, the motions of the residues around proline residues may be confined, making this domain moving with a concerted manner. This speculation is further validated by the result revealed by GNM (see next section). The concerted motion of the B-domain is likely associated with the functions of NpAS. Indeed, two (P230 and P234) out of five proline residues locate nearby the catalytic pocket, especially the P230. As is well known, proline residue can confine the backbone conformation of the adjoining neighbor residues. Here the succeeding residue of the P230 is D231, whose backbone adopt 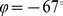 and 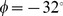, closely resembling the conformation of P230. According to the crystal structures of NpAS in complex with sucrose (PDB accession number: 1MW3 and 1MW0) [13], the sidechain of Asp231 directly contact with the sucrose situated at the alternative sucrose-binding site (SB2) in the B′-domain. It has been described that D231 acts as the most important “geometric lock” responsible for a closed conformation of a highly flexible loop in the B′-domain [67]. Removal of the Asp231 side chain allowed simulation of large movements of this loop using geometric techniques [68].

**Table 4:** The distribution of proline residues among each domain.

	NpAS		DgAS	
Domain	Length	Distribution	Length	Distribution
N	89	4(4.5%)	84	2(2.4%)
A	323	16(5.0%)	331	20(6.0%)
B	76	5(6.6%)	81	6(7.4%)
B′	65	2(3.1%)	69	2(2.9%)
C	75	3(4.0%)	85	6(7.1%)

Concerning on the B-domain of DgAS, its proline proportion is slightly increased to 7.4% owing to an additional proline residue (P219). Unsurprisingly, the B-domain of DgAS still holds the highest proline proportion comparing with the other domains. According to Figure 1, the additional proline residue, i.e. the P219, in the B-domain of DgAS, corresponds with the R226 in NpAS. This discrepancy should be of great interest to protein engineers. On the one hand, R226 of NpAS has been identified that it can limit the binding of maltooligosaccharides, resulting in the accumulation of small products in the medium [69]. The R226A substitution has been proved to be a remarkable mutant that produces as twice as much insoluble glucan as the wild-type NpAS [69]. On the other hand, however, the mutant of R226A has also been proved to be less stable than the WT NpAS. Interestingly, DgAS seems to adopt an alternative way to incorporate stability and specificity together. The P219 of DgAS is much smaller than the R226 of NpAS in size, therefore, may contribute the high stability of the DgAS. Based on the virtual Ala-scan on DgAS, the WT DgAS is indeed more stable than the P219A mutant (Table S2). Except that, we also calculated the free energy changes for the R226P substitution of NpAS (Table S3). According to the result, the folding free energy of the R226P mutant is 0.5 kcal·mol^−1^ lower than that of the WT NpAS. Consequently, substituting R226 with P226 may be a practical way to enhance the thermostability of NpAS while holds or even improves its product specificity.

As to the B′-domain, the proline proportions for the two AS are of little difference. From Table S3, we can see there is only one pair of conserved proline residues in the two AS. The local conformation of D427 of NpAS is quite similar with the P430 of DgAS, thus the P430 is considered as transplantable. As a matter of fact, the folding free energy of the D427P mutant is estimated to be about 1.0 kcal·mol^−1^ lower than that of the WT NpAS according to calculation, thus implicating D427P may elevate the thermostability of NpAS. The V438 of DgAS is corresponding to the P435 of NpAS. According to calculation, substituting V438 with P438 lower the folding free energy of DgAS by 0.5 kcal mol^−1^. This suggests that the thermostability of DgAS could be elevated by using the structural features of NpAS as reference.

The N-domain of NpAS holds 5 proline residues, which is unexpected when comparing with that of the DgAS. Even more surprisingly, one can see that there is only one pair of common proline residues, i.e. the P59 of NpAS and the P54 of DgAS, between the two AS (Figure 1). When scrutinizing the local structures, however, it is found that even the two common proline residues adopt slightly different conformations when superimposing the two AS together. As for the P2 and P17, no corresponding residues are found in DgAS, since the local structures for the two AS are quite different. According to the crystal structures of NpAS (PDB accession number: 1MW3), a possible sucrose binding site (SB3), locating around the _12_LDIYTPEQRAGIE_24_ peptide of the N-domain, is identified [12]. This evidence suggests the N-domain of NpAS may also play a role in the activity of this enzyme. In contrast, based on the comparison among the available crystal structures of NpAS and DgAS, there is no corresponding sucrose binding pocket found around the very positions of DgAS. Although sharing a similar scaffold, the functions of the N-domain of NpAS are probably different with those of the DgAS because of the _16_TPEQRAGI_23_ insertion in NpAS. The P41 of NpAS is closely corresponding to the E36 of DgAS, and this the only match we can identified in structure. Based on calculation, the E36P substitution lower the folding free energy of DgAS by 1.1 kcal·mol^−1^ (Table S3). This is another possible point mutation that possibly enhances the thermostability of DgAS. Besides, the backbone dihedral of N76 of NpAS closely resembles that of the P69 of DgAS, and substituting N76 with P76 results in −1.96 kcal·mol^−1^ changes on the folding free energy. This point mutation may also be considered as a possible thermostability-elevating substitution.

The proline proportion of the C-domains of NpAS and DgAS are quite different. The C-domain of NpAS possesses only 3 proline residues, however, the number increases to 6 for that of DgAS. This suggests the C-domain of DgAS may be intrinsically more stable than that of the NpAS. Unlike the other domains, there is no common proline residue found in the C-domains of the two AS. According to Figure 1, one can see that all proline residues of NpAS have corresponding positions in DgAS. As far as the C-domain of DgAS is concerned, four out of six proline residues can be mapped to the corresponding positions of NpAS. These observations suggest these proline residues are possibly “transplantable” between the corresponding positions of the two AS. This speculation is supported by the results of free energy calculation (Table S3). Among the 4 possible substitutions for NpAS, the T589P mutant is the most promising one, lowering the folding free energy by 2.1 kcal·mol^−1^. Checking the local structure around T589, it is found that this residue is located at the joint point between a β-strand and a short helix, and the near by residues are not tightly packed against it, thus substituting the threonine residue with a proline residue at this very position is probably beneficial to the thermostability of the protein. For the other two positions, i.e. the N560 and the D614, substitution with proline residues at these positions are also thought to be helpful for the elevation of thermostability. As to the N562P mutant, however, a very bad folding free energy has been estimated because of the serious VDW clash. Since the N562 is on the surface of the protein, therefore, the bad VDW clash may be eliminated by the slight adjustment on the local structure. As far as the C-domain of DgAS is concerned, the T601P and the G637P substitutions may be good for the thermostability engineering, whereas the L613 is not suitable to be substituted by the proline residue because of the huge VDW clash (Table S3).

Being the core domain of AS, the sequence of the A-domain is more conserved than those of the N and the C-domains (Figure 1), and so are the proline residues. Within the 16 proline residues of the A-domain of NpAS, 13 of them are conserved between the two AS and the others may also be introduced to the corresponding positions of the DgAS. According to the folding free energy calculation on mutants of DgAS, the D113P substitution is predicted to be stabilized but the R132P and the N354P each are believed to be destabilized owing to bad VDW clashes (Table S3). As far as the DgAS is concerned, 19 proline residues are found in the A-domain, and 13 are conserved and the other 7 may be also reproducible in NpAS. In the light of energy calculation, however, only the N378P, the S502P and the A530P substitutions are predicted to be stabilized (Table S3). Since the A-domain is critical for the catalytic activity of AS, the intrinsic thermostability of this domain is of great importance for the efficiency of the enzyme. Suggested by the higher proline proportion, we believe the A-domain of DgAS is likely more stable than that of NpAS.

### II. Comparison between the Dynamics of the two AS

#### The Global Motion Modes for NpAS and DgAS are Similar

Motions captured by the elastic models of a protein, specifically the first few slowest modes are global in nature and have been reported to capture the biologically and functionally relevant motions of a protein [70], [71], [72]. They are able to represent conformational changes around the native state and allow capturing more energetically accessible structural rearrangements [73]. The slowest modes have also been shown to correlate with the experimental observations of conformational changes of proteins [72], [74], [75].

**Figure 3:**
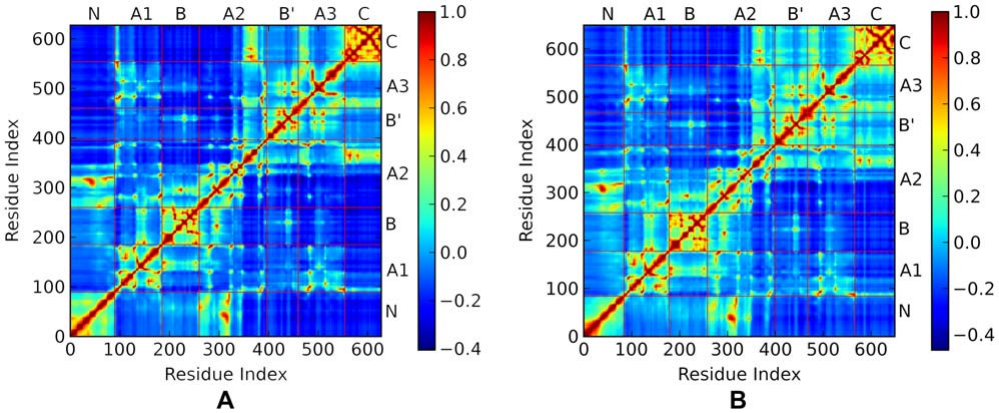
The cross-correlation maps for the first 40 slow modes of NpAS (A) and DgAS (B). The A-domain is decomposed into three parts because of the two insertion domains, i.e. B and B′. A1 part is composed of α1–α2 and β1–β3. A2 part is composed of α3–α6 and β4–β7. A3 part is composed of α7–α8 and β8.

In order to capture the global motions of the two AS, the cross correlation for the slow motions of the two AS were calculated using Eq. (7). Since the modes with low frequency correspond to functional motions and those with high frequency correspond to localized motions, only the slow modes are used to improve the signal/noise ratio. Here the first 40 modes are used for both the NpAS and the DgAS. Altogether, they can explain over 50% of the total motions. The results for NpAS and DgAS are depicted in Figure 3 (A) and (B), respectively. From Figure 3(A), we found the cross correlation map for the first slowest mode of the NpAS can be roughly divided into two regions based on correlations. The first region is constituted by residues from 1 to 341. This region includes the N, B and the major half of the A-domain (α1–α5 and β1–β5). These parts, therefore, may move with a concerted manner since they are topologically connected. The second region is composed of the residues 342 to 628. These residues just constitute the B′, C and the minor half of A-domain (α6–α8 and β6–β8). Probably, the motions of these parts are also weakly coupled together according to the cross correlation map. Besides, it is noticed that the motions of the C-domain are highly concerted, with an average correlation over 0.6. This just reflects the topology of the C-domain, which is purely constituted by tightly packed β-sheets. Obviously, the motions of the first region and the second region of NpAS are negatively correlated according to Figure 3(A), which means the two regions may move along with two different directions. Except these features, we also notice that the residues from 100 to 250, representing the B and a part of the A-domain (α1–α3 and β1–β3) are weakly correlated with the second region, reflecting the topology of the local structure. Although the overall feature of the cross correlation map of DgAS is closely similar with that of the NpAS, it is found that the cross correlation for the local structures within each domain are slightly stronger, suggesting the packing among these local structures is more tight.

**Figure 4:**
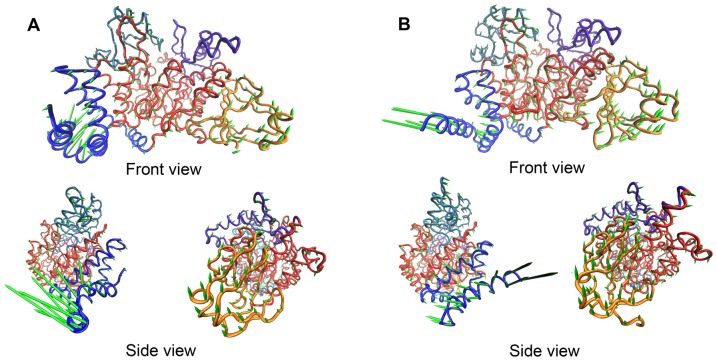
The first slowest motion modes of NpAS (A) and DgAS (B) revealed by the ANM. The length of the cone in each C_α_ atom represents the magnitude of movement and its direction indicates the moving direction.

According to the crystal structures of the two AS, the flexible loops of the B and B′ and A-domains constitutes the geometrical door of the main active site. In order to access the binding pocket, the sucrose molecule has to break through the hindrance formed by these loops. From both the experimental and the calculated B-factors of the two AS (data not shown), we can see that these loops are more flexible than the other parts in the same domain. Based on the cross correlation map (Figure 3(A) and (B)), the loops constituted the door may move alone different directions, therefore, these motions are likely associated with the binding of subtract and releasing of product. To support this assumption, we took advantage the ANM and the first slowest motion modes for the two AS are given by Figure 4(A) and (B), respectively. Apparently, the first slowest modes of the two AS show a typical shear motion in both the thermostable and the mesostable AS according to the results of ANM. Clearly shown by the top view plots for the two AS, the slowest motions of the two proteins can be divided into two parts with nearly opposite directions. The N, B and the major half of A-domain move along one direction, and the C, B′ and the minor half of A-domain moves along the opposite direction. These results are in great accordance with the analyses based on the GNM. With the shear-like motion, the door constituted by the flexible loops of the B, B′ and A-domain may switch between the open and the closed states, thereby possibly facilitating the binding of subtract and the releasing of product.

#### The Difference in Hot Spots Reflect the Difference in the Local Structure

**Figure 5:**
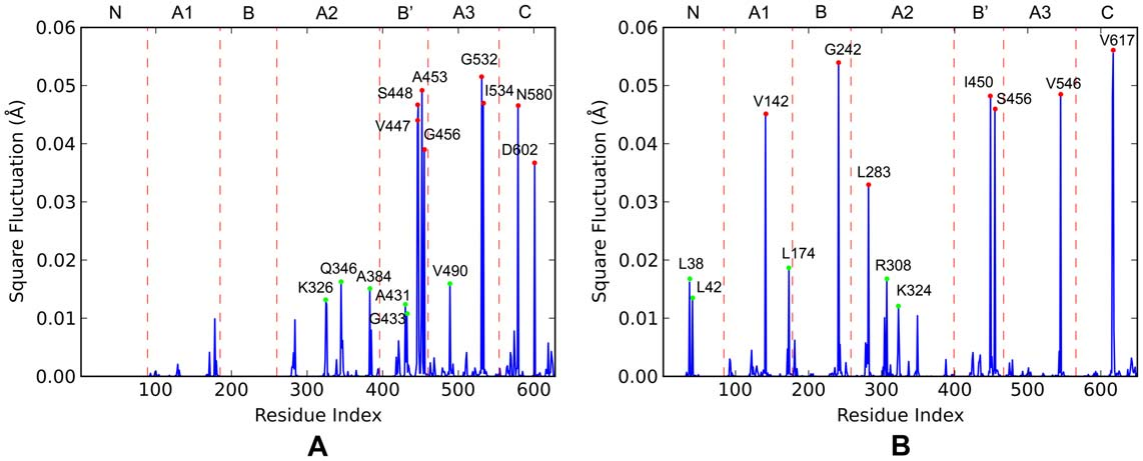
The square fluctuations for the first 10 fastest models of NpAS (A) and DgAS (B). The assuming hot and warm spots are highlighted by red and green dots, respectively.

The fast modes correspond to geometric irregularity in the local structure and the fluctuations associated with fast modes are accompanied by a decrease in entropy larger than that for slow modes [76]. Therefore, residues with higher fluctuations in the fast modes are thought to be kinetically hot spots and are critically important for the stability of the tertiary fold [75], [76], [77]. For the sake of gaining insight into the hot spots of the two AS, the fastest 10 modes of are included into consideration and the results are plotted in Figure 5(A) and (B) for NpAS and DgAS, respectively. Surprisingly, the hot spots revealed by the fast modes for the two AS are quite different, which hints the local structures of the two AS are of significant differences.

In this work, a residue is regarded as a possible hot spot if its square fluctuation (SF) of the fastest 10 modes is larger than 0.03 Å^2^, and is regarded as a possible warm spot if its SF of the in the fastest 10 modes is larger than 0.01 but less than 0.03 Å^2^. According to this criterion, NpAS possesses 8 hot spots and 6 warm spots; DgAS possesses 7 hot spots and 5 warm spots. These spots are marked in Figure 5(A) and (B) with different colors. According to these results, the kinetically important spots of NpAS are predicted to be mainly located at B′ and A (A3 part) and C-domains, however, no hot or warm spots are found in the N-domain. In contrast, the hot/warm spots of DgAS distribute over all domains. Notably, DgAS possesses two warm spots, i.e. L38 and L42, in N-domain. Besides, R308 of DgAS, directly contacting with L38 and L42, is also thought as a hot spot. Based on the structural comparison, it is found that these three residues are conserved in NpAS and DgAS. These residues, however, are not discovered even in the first 15 fastest modes of NpAS (data not shown). By scrutinizing the local structures around these three residues, we found the local structures are of great differences though the overall folding architectures are similar. Further, when investigating the natural contact number of the three residues, it was found that the residues of DgAS possessed more contacts than those of the NpAS. In DgAS, L38, L42 and L308 all have 12 contacts, whereas the corresponding residues of NpAS only have 10, 9 and 11 contacts, respectively. This may partly account for the reason why these resides are recognized as hot or warm spots only in DgAS but not in NpAS. Similarly, the V142 and L283 of DgAS are recognized as hot spots, whereas their counterparts (L149 and M285) in NpAS are not. The G242 in the B-domain of DgAS is predicted to be a hot spot, but the residue in the corresponding position of NpAS (R244) is not. This is because that there is a five-residue-insertion (236IGEGE240) precedes the G242 of DgAS, hence resulting in the local structure of DgAS is quite different from that of NpAS. The other major difference in hot spots resides in the C-domains of the two AS, reflecting the differences in their local structures too.

In spite of these differences, two conserved hot regions are discovered. The first hot region (residues 431–456 in NpAS and residues 434–459 in DgAS) is located at the B′-domain and the second one is located around the long loop connecting β8 and α8 of the A-domain (A3 part). The second region is geometrically close to the first region. Since the loops of B, B′ and A-domain are considered to be related with the functions of AS, the stability of these domains would be of great importance to the activity of the enzyme. Indeed, many functionally important residues of NpAS, such as R446, D394, D393, are in the close vicinity of these hot spots. Taking all of these results into consideration, it is speculate these conserved hot spot residues may act as folding nucleus, and thereby are critical for the activity of AS. This speculation is supported by results revealed by the iterative GNM (see the following section).

### III. The Unfolding Property of the Two AS

#### The Overall Unfolding properties of the two AS

**Figure 6:**
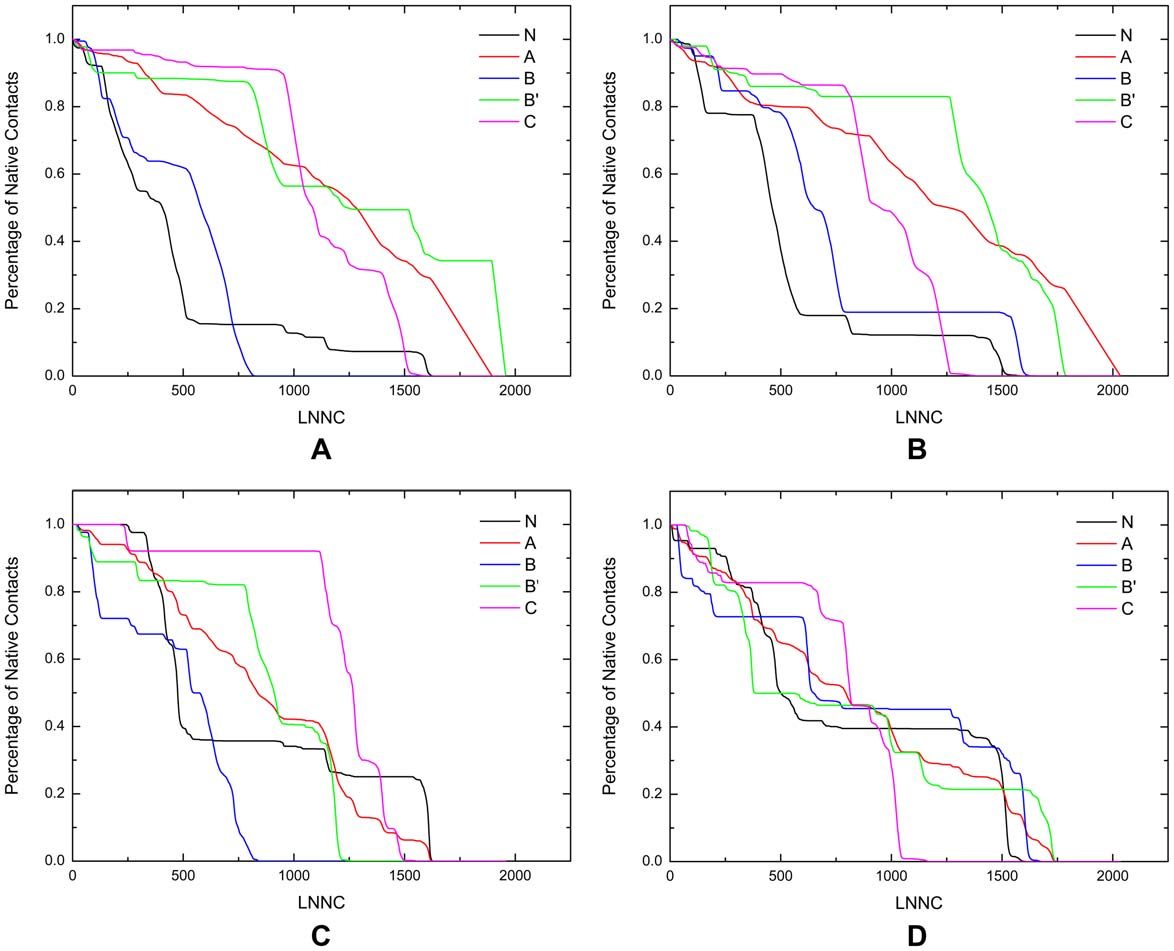
The unfolding curves for each domains of NpAS and DgAS. Here, the unfolding curves for the intra domain contacts of NpAS and DgAS are displayed in (A) and (B), and those for the inter domain contacts of NpAS and DgAS are shown in (C) and (D), respectively.

Understanding the unfolding process of a protein is of great importance for the rational design of kinetic stability. Kinetic stability, in the context discussed here, is a measure of how rapidly a protein unfolds. It is a particularly important consideration for proteins that unfold very slowly or denature irreversibly, such as aggregation or proteolytic degradation. In cases such as these, it is not the free energy difference between the folded and unfolded state that is important. That will only influence the equilibrium between the folded and unfolded state, however, it is not an equilibrium process anymore. The important thing is the free energy difference between the folded and the transition states (activation energy), for it is the magnitude of this difference that determines the rate of unfolding and hence inactivation. Clearly, it would be very helpful for the rational design if we could gain some information of the unfolding sequence of the two AS ahead of the time consuming design and validation cycles. With the priori knowledge of the unfolding properties of the two AS, one can reinforce the weak parts specifically, thus slowing down the unfolding paces and hence the inactivation tendency. In order to figure out the unbinding processes of the two AS, the iterative GNM was performed as described in the method section. For the sake of clarity, the connections of between nodes are decomposed into two types: the intra-domain connections and the inter-domain connections. Obviously, the broken of the intra-domain connections in one domain mainly describe the unfolding of the domain itself, say, the unfolding of secondary structures. In contrast, the broken of the inter-domain connections stand for the unfolding around the interface between domains. Here, the unfolding curves for each domain of NpAS and DgAS are displayed in Figure 6(A) and (B), and those for the interfaces between domains in NpAS and DgAS are shown in Figure 6(C) and (D), respectively. The disconnection order for the intra and inter domain connections together describe the whole unfolding process of each domain.

Clearly, the overall unfolding property of NpAS is quite different form that of DgAS based on the comparison between Figure 6(A)/(C) and (B)/(D). Although the shape of the unfolding curves for the intra-domain contacts of the two AS are of somewhat similarity, the specific unfolding order of each domain is not exactly the same. Furthermore, the unfolding curves for the inter-domain contacts of the two AS are totally different. In NpAS, the inter-domain contacts for all domains but the A-domain were broken rapidly at some critical points, however, the inter-domain connections for all domains of DgAS broke with a relatively smooth manner. These evidences may account for the differences in the heat resistance of the two AS. In order to further explore this problem, the unfolding curve for each domain is discussed in great detail in the following text.

According to Figure 6(A) and (C), the unfolding process of NpAS began with the collapse of the N-domain. This is not surprising since many studies have shown that protein is prone to unfold from the N-terminal [42], [43], [78]. Firstly, the inner connections of the N-domain started to disconnect at the very beginning of the unfolding process, however, the connections between the N and A-domain remained untouched until around the step of LNNC = 330. After that, the connections between the N and A-domain began to rupture quickly within 200 steps. Around the 514th step, only 36% inter connections between the domains are left. In the next place, the secondary structures of the N-domain continued to collapse quickly, while the inter connections with the A-domain broke very slowly. The N-domain became almost fully unfolded around the 550th steps, at which the many domains even did not begin to show any remarkable unfolding. Next, the residual secondary structures of the N-domain unfolded with a very slow pace, and the connections between the N and A-domain kept stable till the end of the entire unfolding process of NpAS. According to the comparison between Figure 6(A)/(C) and (B)/(D), it is found the unfolding process for the N-domain of DgAS essentially resembles that for the N-domain of NpAS.

To explain why the two AS all began to unfold from the N-domain, we calculated the native contact number for each domain of the two AS, and the strength of the contact in each domain can be roughly measured by the value of NCPR (number of contact per residue, see Table 5). Evidently, the N-domains of the two AS possess the least NCPR among all domains. From the aspect of practical applications, therefore, it would be helpful to slow down the inactivation of the NpAS if we can enhance the connections in the N-domain and those between the N and A-domain.

**Table 5:** The Number of Contact per Residue (NCPR) for each domain of NpAS and DgAS.

	NpAS			DgAS		
	Num Con	Num Res	NCPR	Num Con	Num Res	NCPR
N	256	89	2.88	245	84	2.91
B	221	76	2.91	244	81	3.01
B′	208	65	3.20	228	69	3.30
C	239	75	3.19	261	85	3.07
A	1033	323	3.20	1056	331	3.19

Being the core-domain of AS, the A-domain was thought to be stable, and hence to unfold at later stages. From Table 5, one can see that the A-domains of the two AS indeed have big NCPR. According to the results of the iterative GNM, however, we found the A-domains of NpAS and DgAS began to collapse at an early stage too. Unlike any other domain, whose unfolding is quickly, the whole unfolding process of the A-domains of the two AS are relatively smooth, reflecting the unfolding took place gradually. The A-domain is located at the center of the enzyme, and all other domains directly contact with it, therefore, the unfolding of other domains may inevitably affect its topology structure and hence its unfolding property. Consequently, it is suggested that reinforcing the other domains may be also helpful for the stabilization of the A-domain.

**Figure 7:**
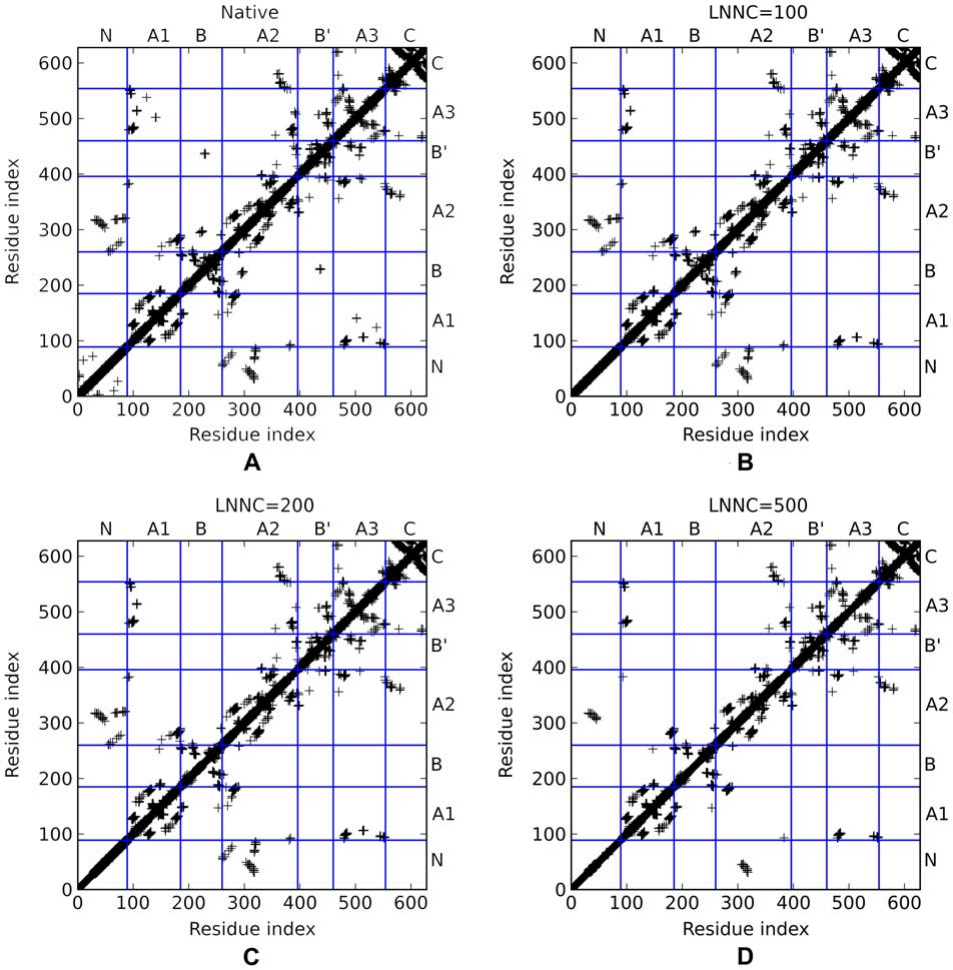
The contact maps for the native (A), LNNC = 100 (B), 200 (C) and 500 (C) states of NpAS (D). Each contact is represented by a “+” mark.

Although with similar size, B and B′-domain of NpAS each possess distinct fold, and so are those domains of DgAS. The B-domain is composed of a four-strand β-sheet which is sandwiched between two short α-helixes. The B′-domain, however, has a relatively simple fold. The B and B′-domain of NpAS exhibit sheerly different unfolding curves according to Figure 6(A) and (C). Depicted by these two figures, the intra connections of the B-domain were quickly broken, accompanied by the rapid broken of the inter-domain connections between B and other domains. On the contrary, the B′-domain of NpAS did not show any obvious unfolding before the 530th step. After that, however, this domain suddenly began to unfold with high speed. During the unfolding process, the disconnection of the intra and inter-domain contacts were roughly synchronic. In DgAS, the B and B′-domain also show different unfolding processes. Over 80% intra-domain contacts of the B-domain were quickly broken within the first 800 steps, and then the residual intra connections were slowly broken in the following steps. This is consistent with the results revealed by Figure 5(B), where the residues around G242 are assumed to be highly constrained. In contrast, the unfolding for the interfaces between the B-domain and other domains was gradual and no sudden decrease in inter-domain connections was observed. The B′-domain of DgAS itself also began to unfold from a very late stage as shown by Figure 6(B), whereas the inter-domain connections of the B′ domain gradually collapsed from the very beginning to the end of the entire unfolding process (Figure 7(D)). Actually the B′-domain was the last domain that began to unfold in DgAS. This is in accordance with the results revealed by the hot spots analyses (see the previous section).

The C-domain of NpAS was the last domain that began to unfold. Most of the connections of the C-domain, no matter the intra-domain or the inter-domain ones, were kept untouched for a long period, and then the domain began to rapidly collapse. Although the shape of the unfolding curve for the C-domain of DgAS is roughly similar with that of NpAS, the unfolding property of this very domain is quit different with its counterpart of NpAS. Clearly, the C-domain of DgAS began to unfold ahead of the unfolding of B′-domain. According to Figure 6(D), the inter domain contacts for all but the C-domain of DgAS were broken gradually, suggesting the N, B and B′-domain of DgAS are coupled well with the A-domain of DgAS. According to calculation, nevertheless, the number of the native inter-domain contacts for each domain in DgAS is comparable with that of NpAS (data not shown), which suggests the topology of the interface between each domain of DgAS is similar with that of NpAS. Based on Table 5, it is found that the NCPR for the N, B and B′-domains of DgAS each is bigger than that of NpAS, which indicates the topology structures for these domains of DgAS are more compact than those of NpAS. Since with stronger topology structures, it was speculated that the unfolding paces of these domains of DgAS might be slowed down. As to the C-domain of DgAS, its NCPR is smaller than its counterpart's of NpAS. This may partially explain why the C-domain of DgAS began to unfold before the B′-domain. Combining these observations together, we believe that the topological property of DgAS is better than that of NpAS thanks to its higher NCPR.

#### Identification of the Weak Regions of the Two AS

**Figure 8:**
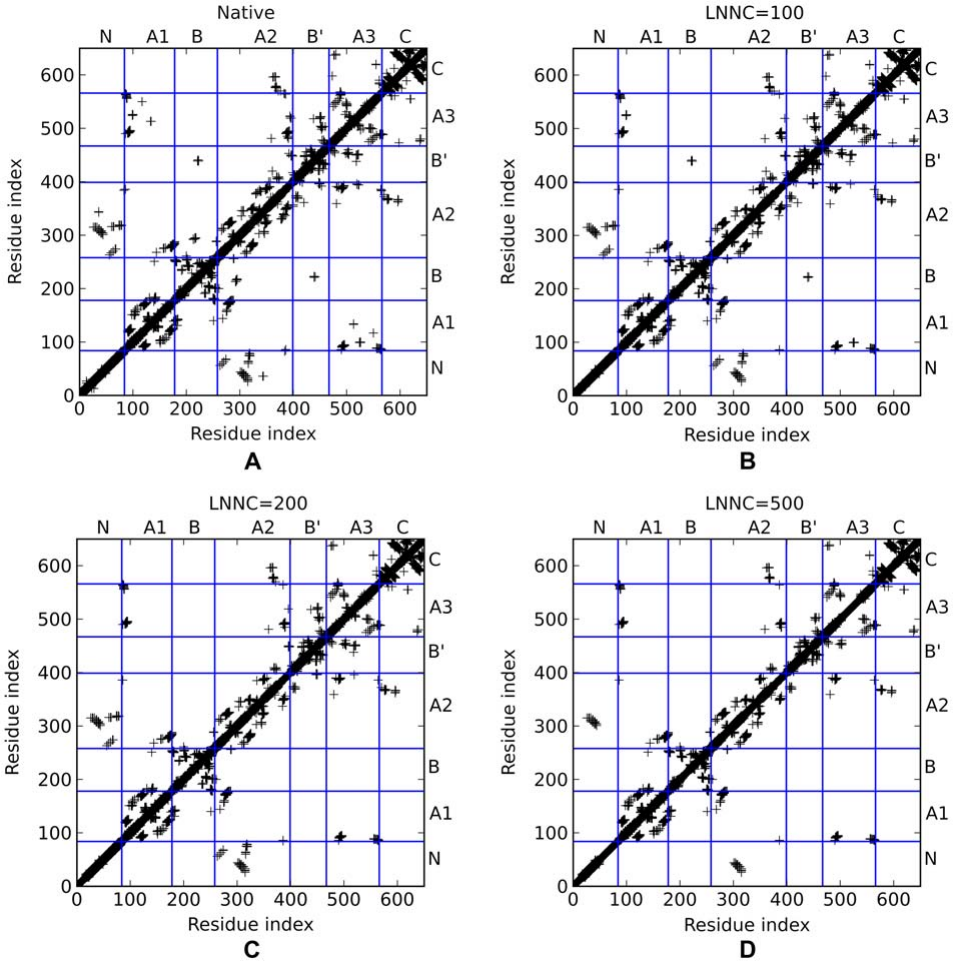
The contact maps for the native (A), LNNC = 100 (B), 200 (C) and 500 (C) states of DgAS (D). Each contact is represented by a “+” mark.

**Figure 9:**
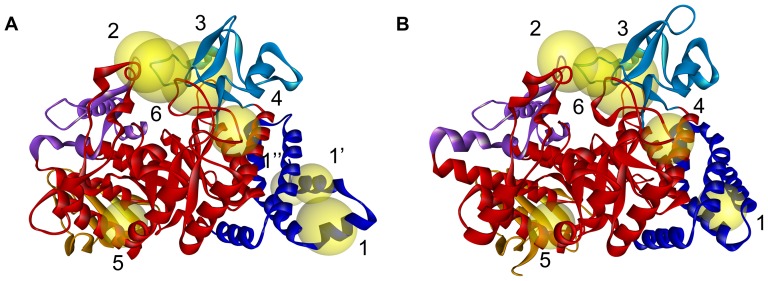
Weak regions revealed by the iterative GNM for NpAS (A) and DgAS (B). Weak regions are represented by yellow spheres. The center of each sphere locates at the geological center of each weak region and the diameter the sphere roughly reflects the size of the weak region.

Although the unfolding curves can tell the overall unfolding sequence for each domain, it can not display the details of the unfolding process. In order to monitor the details in the unfolding process of the two AS, the contact map was plot and saved every 10 steps. Since there are about two hundred plots for the whole unfolding process of each AS, these plots are mainly submitted to the supplementary materials for the sake of saving space. Only the contact maps for the native states and the loss-number-of-native-contact (LNNC) = 100, 200 and 500 states of NpAS and DgAS are given by Figure 7(A)–(D) and Figure 8(A)–(D), respectively. The full unfolding processes for the two AS are given in the supplementary materials (Figure S1 and S2). Here we arbitrarily define a contact as the weak if it was broken within 500 steps, i.e. breaking approximately 20% of the total contacts. According to these figures, the kinetically weak regions of the two AS were identified, and the results are displayed in Figure 9(A) and (B).

Since the contacts within each secondary structure are relatively strong than those between two close secondary structures, most weak regions of each domain are found to be located around the interface between two independent secondary structures. Understanding the information about these weak regions ahead of time will be quite helpful for our following design and validation cycles. The kinetically weak regions of NpAS and DgAS are depicted by Figure 9(A) and (B). Being the start point of the unfolding, the contacts associated with the N-domain of NpAS are of course apt to be broken. Particularly, the contacts among α-helixes of the N-domain, such as D28-S73 (region 1), I11-A66 (region 1′) and S4-M36/D37/F40 (region 1″), were disconnected at the very beginning of the unfolding process. For DgAS, the topology of the N-domain is slightly different from that of NpAS, and the weak region is found to be around the V14-L28 (region 1). Additionally, the interface between the N- and the C-domain of both of the two AS are also vulnerable, and thereby could be a suitable region for engineering.

According to the contact map of the native state of NpAS (Figure 7(A)), it is found that there are only a few connections between the B and B′-domain. The involving residues are F229, P230 and D231 in the B-domain as well as Q437, N439 in the B′-domain (region 2). These connections were broken at the very beginning of the unfolding process as well. As is mentioned previously, D231 acts as acts as the most important “geometric lock” of NpAS, therefore, the contacts between D231 and the residues around it are likely critical for the functions of the enzyme. Since these critical connections breaks at a very early stage (around LNNC = 80), implicating NpAS becomes inactive at the beginning of the unfolding process. This may partly account for the reason why NpAS is notoriously susceptible to heat. In contrast, these connections in DgAS disappear around LNNC = 190, thereby reflecting its higher heat resistance. Comparing the local structures of DgAS with those of NpAS, it is found the D224 of DgAS, stays closer to the loop of B′-domain than its counterpart (D231) does in NpAS. The slight differences in the local structures may result in the slight differences in the unfolding properties of the two AS, and thereby bringing on the different heat resistances.

Another weak region of NpAS is located around the interface between the B-domain and the A-domain (A2 part). In the native state, the 221YDRTLR226 peptide of the B-domain direct interacts with the 295MGTSC299 peptide of the A-domain (region 3), however, these connects disappeared around 110th step mainly because of the deformation of the B-domain itself. The situation in DgAS is similar and the corresponding weak region is around 214FEATLP219 peptide of B-domain and the 293LETDC297 peptide of A-domain (region 3). As the core domain, the interfaces among secondary structures in the A-domain of NpAS are relatively strong, nonetheless, three exceptional regions were still found (Figure 8), and the involved weak contacts are Y152-E271 (region 4), L125-R539 (region 5) and G141/K142-N503 (region 6). In DgAS, the corresponding regions are also thought to be apt to unfolding at early stages.

## Discussion

In this work, the structure, dynamics and unfolding properties of the two AS were thoroughly investigated for the sake of explaining the reason why DgAS is more stable than NpAS and revealing novel insight into rational design on thermostability. Based on the results addressed above, there are two main factors that contribute to the superior thermostability of DgAS. Firstly, DgAS holds some good features in structure that may make positive contributions to the thermostability. Secondly, the contacts among residues of DgAS are thought to be topologically more compact than those of NpAS owing to its higher NCPR value.

Among these merits in structure, the number of H-bonds, salt-bridges, and the proline proportion are of great importance to the thermostability, especially the proline proportion. DgAS possesses more backbone-backbone H-bonds and salt-bridges than NpAS does, suggesting the interactions among residues of DgAS are stronger. Owing to the high proline proportion, the conformational entropy of DgAS in both of the folded and the unfolded states is highly decreased, thus stabilizing the enzyme. When investigating the distribution of proline residues over each domain of NpAS and DgAS (Table 3), however, it is found that only the A, B and C-domains of DgAS hold more proline residues than those of NpAS. The B′-domain of DgAS holds the same number of proline residues as that of NpAS As to the N-domain of DgAS, nonetheless, its proline proportion is less than that of the N-domain of NpAS. These results, at first glance, seems to be inconsistent with the fact that DgAS is much more than NpAS, yet this paradox will be clear when taking the NCPR value for each domain into consideration. Combining the results of Table 3 and table 5 together, an interesting phenomenon is discovered: the domain with relatively small NCPR, such as the A and C-domain, in DgAS tends to hold more proline residues than its counterpart in NpAS does. What's more, the B and B′-domain of DgAS has a much bigger NCPR than their counterpart of NpAS. Consequently, these results just suggest that the reason why DgAS is more stable than NpAS is owing to not only its structural merits but also its merits in topology.

Unlike other researches that focus on the structural differences between the thermophilic and the mesophilic proteins, in the current study, we also explored the dynamics and unfolding property of the two AS. According to the results of GNM and ANM, we have found that the two AS could exhibit a shear-like motion, which is probably associated with their functions. What is more, with the discovery of the unfolding pathway of the two AS, we can focus on the weak regions, and hence designing more appropriate mutations for the sake of thermostability engineering. Based on the comparisons between the structures of the two AS, the appropriate positions for proline substitution have been revealed and discussed above. In total, there are 13 and 8 possible positions for proline substitutions in NpAS and DgAS, respectively. Among the 13 possible substitutions in NpAS, 9 of them were predicated to be stabilized and the others are believed to be destabilized because of large steric conflicts (Table S3). Checking the unfolding order of NpAS, it was found that most of them were predicted to unfold at early stages. Owing to these results, we speculate that substituting these residues with proline residues may be helpful for the engineering on thermostability. Among the 8 possible substitutions in DgAS, only 3 of them were predicted to be stabilized, 3 were neutral, and the left 2 were believed to be destabilized on the basis of the free energy changes (Table S3). Although the contacts among E36 and other residues were broken at very late stages, the connections formed around the residues by its both sides and the others began to be ruptured at the very beginning of the unfolding process. For example, the connections between D37 and H344/G345 were disconnected around the 10th step. Besides, the residue just located before E36 is G35. According to Figure 4 (B), it is found that the 35GED37 peptide is quite flexible. Substituting Glu with Pro in position 36 probably restrain the mobility of the local structure, thus may slow down the unfolding process. The heat resistance of the D113P mutant, however, may be no better than that of the WT enzyme since all the around residues are stable. As to the G637P mutant, it may be actually stabilized because of the large VDW clash shown by energy decomposition (data not show). In order to find other possible positions which are suitable for proline residues, one can resort to the virtual Pro-scan method, i.e. substituting each residue of the target protein by the proline residue and calculating the free energy changes.

In conclusion, the factors that constitute the superior thermostability of DgAS have been discovered through a combinational method of structural and topology analyses. These factors can at least partially explain the reason why DgAS is much more thermostable than NpAS, which may be helpful for the rational design of mutants with higher thermostability. Besides, the dynamics and unfolding property of the two AS have been discussed in great details, and hopefully these discoveries can be used as guides for our future work on rational design.

## Supporting Information

Figure S1 **The detailed unfolding pathway for NpAS.**
(TIF)

Figure S2 **The detailed unfolding pathway for DgAS.**
(TIF)

Table S1 **The Ala-scan for glycine residues of NpAS and DgAS.**
(DOC)

Table S2 **The Ala-scan for proline residues of NpAS and DgAS.**
(DOC)

Table S3 **The free energy calculation for the proline residues of NpAS and DgAS.**
(DOC)

## References

[1] Henrissat B (1991). A classification of glycosyl hydrolases based on amino acid sequence similarities.. Biochem J.

[2] Henrissat B, Davies G (1997). Structural and sequence-based classification of glycoside hydrolases.. Current Opinion in Structural Biology.

[3] Cantarel BL, Coutinho PM, Rancurel C, Bernard T, Lombard V (2009). The Carbohydrate-Active EnZymes database (CAZy): an expert resource for Glycogenomics.. Nucleic Acids Research.

[4] Hehre EJ, Hamilton DM (1946). Bacterial synthesis of an amylopectin-like polysaccharide from sucrose.. J Biol Chem.

[5] MacKenzie CR, McDonald IJ, Johnson KG (1978). Glycogen metabolism in the genus Neisseria: synthesis from sucrose by amylosucrase.. Can J Microbiol.

[6] Riou JY, Guibourdenche M, Popoff MY (1983). A new taxon in the genus Neisseria.. Ann Microbiol (Paris).

[7] Potocki de Montalk G, Remaud-Simeon M, Willemot R-M, Sarçabal P, Planchot V (2000). Amylosucrase from Neisseria polysaccharea: novel catalytic properties.. FEBS Letters.

[8] Pizzut-Serin S, Potocki-Véronèse G, van der Veen BA, Albenne C, Monsan P (2005). Characterisation of a novel amylosucrase from Deinococcus radiodurans.. FEBS Letters.

[9] Emond S, Mondeil S, Jaziri K, André I, Monsan P (2008). Cloning, purification and characterization of a thermostable amylosucrase from Deinococcus geothermalis.. FEMS Microbiology Letters.

[10] Ha SJ, Seo DH, Jung JH, Cha J, Kim TJ (2009). Molecular cloning and functional expression of a new amylosucrase from Alteromonas macleodii.. Biosci Biotechnol Biochem.

[11] Skov LK, Mirza O, Henriksen A, Potocki de Montalk G, Remaud-Simeon M (2000). Crystallization and preliminary X-ray studies of recombinant amylosucrase from Neisseria polysaccharea.. Acta Crystallogr D Biol Crystallogr.

[12] Skov LK, Mirza O, Henriksen A, De Montalk GP, Remaud-Simeon M (2001). Amylosucrase, a glucan-synthesizing enzyme from the alpha-amylase family.. J Biol Chem.

[13] Skov LK, Mirza O, Sprogoe D, Dar I, Remaud-Simeon M (2002). Oligosaccharide and sucrose complexes of amylosucrase. Structural implications for the polymerase activity.. J Biol Chem.

[14] Jensen MH, Mirza O, Albenne C, Remaud-Simeon M, Monsan P (2004). Crystal structure of the covalent intermediate of amylosucrase from Neisseria polysaccharea.. Biochemistry.

[15] Hehre EJ (1949). Synthesis of a polysaccharide of the starch-glycogen class from sucrose by a cell free, bacterial enzyme system.. J Biol Chem.

[16] Potocki de Montalk G, Remaud-Simeon M, Willemot RM, Monsan P (2000). Characterisation of the activator effect of glycogen on amylosucrase from Neisseria polysaccharea.. FEMS Microbiol Lett.

[17] Preiss J, Ozbun JL, Hawker JS, Greenberg E, Lammel C (1973). ADPG synthetase and ADPG- -glucan 4-glucosyl transferase: enzymes involved in bacterial glycogen and plant starch synthesis.. Ann N Y Acad Sci.

[18] De Montalk GP, Remaud-Simeon M, Willemot RM, Planchot V, Monsan P (1999). Sequence analysis of the gene encoding amylosucrase from Neisseria polysaccharea and characterization of the recombinant enzyme.. J Bacteriol.

[19] Emond S, Andre I, Jaziri K, Potocki-Veronese G, Mondon P (2008). Combinatorial engineering to enhance thermostability of amylosucrase.. Protein Sci.

[20] Lehmann M, Wyss M (2001). Engineering proteins for thermostability: the use of sequence alignments versus rational design and directed evolution.. Curr Opin Biotechnol.

[21] Chan CH, Liang HK, Hsiao NW, Ko MT, Lyu PC (2004). Relationship between local structural entropy and protein thermostability.. Proteins.

[22] Bae E, Bannen RM, Phillips GN (2008). Bioinformatic method for protein thermal stabilization by structural entropy optimization.. Proc Natl Acad Sci U S A.

[23] Liu M, Sun T, Hu J, Chen W, Wang C (2008). Study on the mechanism of the BtuF periplasmic-binding protein for vitamin B12.. Biophys Chem.

[24] Liu M, Su JG, Kong R, Sun TG, Tan JJ (2008). Molecular dynamics simulations of the bacterial periplasmic heme binding proteins ShuT and PhuT.. Biophys Chem.

[25] Liu M, Cong XJ, Li P, Tan JJ, Chen WZ (2009). Study on the inhibitory mechanism and binding mode of the hydroxycoumarin compound NSC158393 to HIV-1 integrase by molecular modeling.. Biopolymers.

[26] Bahar I, Rader AJ (2005). Coarse-grained normal mode analysis in structural biology.. Curr Opin Struct Biol.

[27] Ma J (2005). Usefulness and limitations of normal mode analysis in modeling dynamics of biomolecular complexes.. Structure.

[28] Ming D, Kong Y, Lambert MA, Huang Z, Ma J (2002). How to describe protein motion without amino acid sequence and atomic coordinates.. Proc Natl Acad Sci U S A.

[29] Bahar I, Atilgan AR, Erman B (1997). Direct evaluation of thermal fluctuations in proteins using a single-parameter harmonic potential.. Fold Des.

[30] Haliloglu T, Bahar I, Erman B (1997). Gaussian Dynamics of Folded Proteins.. Physical Review Letters.

[31] Tirion MM (1996). Large Amplitude Elastic Motions in Proteins from a Single-Parameter, Atomic Analysis.. Phys Rev Lett.

[32] Atilgan AR, Durell SR, Jernigan RL, Demirel MC, Keskin O (2001). Anisotropy of fluctuation dynamics of proteins with an elastic network model.. Biophys J.

[33] Hinsen K, Kneller G (1999). A simplified force field for describing vibrational protein dynamics over the whole frequency range.. The Journal of Chemical Physics.

[34] Tama F, Sanejouand YH (2001). Conformational change of proteins arising from normal mode calculations.. Protein Eng.

[35] Li G, Cui Q (2002). A coarse-grained normal mode approach for macromolecules: an efficient implementation and application to Ca(2+)-ATPase.. Biophys J.

[36] Su JG, Jiao X, Sun TG, Li CH, Chen WZ (2007). Analysis of domain movements in glutamine-binding protein with simple models.. Biophys J.

[37] Yennamalli RM, Wolt JD, Sen TZ (2011). Dynamics of endoglucanase catalytic domains: implications towards thermostability.. J Biomol Struct Dyn.

[38] Bahar I, Erman B, Jernigan RL, Atilgan AR, Covell DG (1999). Collective motions in HIV-1 reverse transcriptase: examination of flexibility and enzyme function.. J Mol Biol.

[39] Temiz NA, Bahar I (2002). Inhibitor binding alters the directions of domain motions in HIV-1 reverse transcriptase.. Proteins.

[40] Isin B, Doruker P, Bahar I (2002). Functional motions of influenza virus hemagglutinin: a structure-based analytical approach.. Biophys J.

[41] Wang Y, Rader AJ, Bahar I, Jernigan RL (2004). Global ribosome motions revealed with elastic network model.. J Struct Biol.

[42] Su JG, Li CH, Hao R, Chen WZ, Wang CX (2008). Protein unfolding behavior studied by elastic network model.. Biophys J.

[43] Su JG, Xu XJ, Li CH, Chen WZ, Wang CX (2011). An analysis of the influence of protein intrinsic dynamical properties on its thermal unfolding behavior.. J Biomol Struct Dyn.

[44] Berman HM, Westbrook J, Feng Z, Gilliland G, Bhat TN (2000). The Protein Data Bank.. Nucleic Acids Res.

[45] Guerin F, Barbe S, Pizzut-Serin S, Potocki-Veronese G, Guieysse D (2012). Structural investigation of the thermostability and product specificity of amylosucrase from the bacterium Deinococcus geothermalis.. J Biol Chem.

[46] Sali A, Blundell TL (1993). Comparative protein modelling by satisfaction of spatial restraints.. J Mol Biol.

[47] Fiser A, Do RK, Sali A (2000). Modeling of loops in protein structures.. Protein Sci.

[48] Marti-Renom MA, Stuart AC, Fiser A, Sanchez R, Melo F (2000). Comparative protein structure modeling of genes and genomes.. Annu Rev Biophys Biomol Struct.

[49] Eswar N, Webb B, Marti-Renom MA, Madhusudhan MS, Eramian D (2006). Comparative protein structure modeling using Modeller.. Curr Protoc Bioinformatics Chapter.

[50] Phillips JC, Braun R, Wang W, Gumbart J, Tajkhorshid E (2005). Scalable molecular dynamics with NAMD.. J Comput Chem.

[51] Miyazawa S, Jernigan RL (1985). Estimation of effective interresidue contact energies from protein crystal structures: quasi-chemical approximation.. Macromolecules.

[52] Bahar I, Jernigan RL (1997). Inter-residue potentials in globular proteins and the dominance of highly specific hydrophilic interactions at close separation.. Journal of Molecular Biology.

[53] Jernigan RL, Demirel MC, Bahar I (1999). Relating structure to function through the dominant slow modes of motion of DNA topoisomerase II.. International Journal of Quantum Chemistry.

[54] Kundu S, Melton JS, Sorensen DC, Phillips GN (2002). Dynamics of proteins in crystals: comparison of experiment with simple models.. Biophys J.

[55] Chennubhotla C, Rader AJ, Yang LW, Bahar I (2005). Elastic network models for understanding biomolecular machinery: from enzymes to supramolecular assemblies.. Phys Biol.

[56] Guerois R, Nielsen JE, Serrano L (2002). Predicting Changes in the Stability of Proteins and Protein Complexes: A Study of More Than 1000 Mutations.. Journal of Molecular Biology.

[57] Schymkowitz JWH, Rousseau F, Martins IC, Ferkinghoff-Borg J, Stricher F (2005). Prediction of water and metal binding sites and their affinities by using the Fold-X force field.. Proceedings of the National Academy of Sciences of the United States of America.

[58] Schymkowitz J, Borg J, Stricher F, Nys R, Rousseau F (2005). The FoldX web server: an online force field.. Nucleic Acids Research.

[59] Petukhov M, Cregut D, Soares CM, Serrano L (1999). Local water bridges and protein conformational stability.. Protein Sci.

[60] Munoz V, Serrano L (1994). Intrinsic secondary structure propensities of the amino acids, using statistical phi-psi matrices: comparison with experimental scales.. Proteins.

[61] Abagyan R, Totrov M (1994). Biased probability Monte Carlo conformational searches and electrostatic calculations for peptides and proteins.. J Mol Biol.

[62] Potapov V, Cohen M, Schreiber G (2009). Assessing computational methods for predicting protein stability upon mutation: good on average but not in the details.. Protein Engineering Design and Selection.

[63] Guex N, Peitsch MC (1997). SWISS-MODEL and the Swiss-PdbViewer: an environment for comparative protein modeling.. Electrophoresis.

[64] Hendsch ZS, Tidor B (1994). Do salt bridges stabilize proteins? A continuum electrostatic analysis.. Protein Sci.

[65] Némethy G, Leach SJ, Scheraga HA (1966). The Influence of Amino Acid Side Chains on the Free Energy of Helix-Coil Transitions.. The Journal of Physical Chemistry.

[66] Matthews BW, Nicholson H, Becktel WJ (1987). Enhanced protein thermostability from site-directed mutations that decrease the entropy of unfolding.. Proceedings of the National Academy of Sciences.

[67] van der Veen BA, Skov LK, Potocki-Veronese G, Gajhede M, Monsan P (2006). Increased amylosucrase activity and specificity, and identification of regions important for activity, specificity and stability through molecular evolution.. FEBS J.

[68] Cortes J, Simeon T, Remaud-Simeon M, Tran V (2004). Geometric algorithms for the conformational analysis of long protein loops.. J Comput Chem.

[69] Albenne C, Skov LK, Mirza O, Gajhede M, Feller G (2004). Molecular basis of the amylose-like polymer formation catalyzed by Neisseria polysaccharea amylosucrase.. J Biol Chem.

[70] Brooks B, Karplus M (1983). Harmonic dynamics of proteins: normal modes and fluctuations in bovine pancreatic trypsin inhibitor.. Proc Natl Acad Sci U S A.

[71] Keskin O, Durell SR, Bahar I, Jernigan RL, Covell DG (2002). Relating molecular flexibility to function: a case study of tubulin.. Biophys J.

[72] Xu C, Tobi D, Bahar I (2003). Allosteric changes in protein structure computed by a simple mechanical model: hemoglobin T<–>R2 transition.. J Mol Biol.

[73] Sluis-Cremer N, Temiz NA, Bahar I (2004). Conformational changes in HIV-1 reverse transcriptase induced by nonnucleoside reverse transcriptase inhibitor binding.. Curr HIV Res.

[74] Loris R, Langhorst U, De Vos S, Decanniere K, Bouckaert J (1999). Conserved water molecules in a large family of microbial ribonucleases.. Proteins.

[75] Demirel MC, Atilgan AR, Jernigan RL, Erman B, Bahar I (1998). Identification of kinetically hot residues in proteins.. Protein Sci.

[76] Bahar I, Atilgan AR, Demirel MC, Erman B (1998). Vibrational Dynamics of Folded Proteins: Significance of Slow and Fast Motions in Relation to Function and Stability.. Physical Review Letters.

[77] Micheletti C, Carloni P, Maritan A (2004). Accurate and efficient description of protein vibrational dynamics: comparing molecular dynamics and Gaussian models.. Proteins.

[78] Xu X, Su J, Chen W, Wang C (2011). Thermal stability and unfolding pathways of Sso7d and its mutant F31A: insight from molecular dynamics simulation.. J Biomol Struct Dyn.

