# Supplementary material for: Insight into the Structure, Dynamics and the Unfolding Property of Amylosucrases: Implications of Rational Engineering on Thermostability
Source: PLoS One. 2012 Jul 6;7(7):e40441. doi: 10.1371/journal.pone.0040441 (PMC3391273; doi:10.1371/journal.pone.0040441)
Supplement: Table S1 — The Ala-scan for glycine residues of NpAS and DgAS. (DOC) [file pone.0040441.s003.doc]

Table S1 The Ala-scan for glycine residues of NpAS and DgAS

| NpAS | | DgAS | |
| --- | --- | --- | --- |
| Residue | ΔΔG (kcal·mol^-1^) | Residue | ΔΔG (kcal·mol^-1^) |
| GLY22 | -0.32 | GLY35 | -0.39 |
| GLY52 | 2.80 | GLY47 | 3.30 |
| GLY100 | 3.91 | GLY55 | -0.05 |
| GLY101 | -0.32 | GLY93 | 0.70 |
| GLY110 | 1.87 | GLY103 | 2.60 |
| GLY114 | -0.13 | GLY107 | 2.35 |
| GLY126 | 4.23 | GLY117 | -0.03 |
| GLY141 | 1.41 | GLY119 | 2.29 |
| GLY145 | 1.24 | GLY134 | 2.40 |
| GLY146 | 4.14 | GLY138 | 3.18 |
| GLY160 | 4.25 | GLY139 | 4.58 |
| GLY163 | -0.69 | GLY153 | 4.42 |
| GLY176 | 2.51 | GLY167 | -0.23 |
| GLY201 | 2.24 | GLY169 | 3.56 |
| GLY235 | 5.17 | GLY194 | 2.77 |
| GLY236 | -0.01 | GLY210 | 1.79 |
| GLY243 | 3.13 | GLY228 | 3.30 |
| GLY270 | -1.08 | GLY237 | 2.66 |
| GLY279 | 4.58 | GLY239 | 1.33 |
| GLY296 | 2.58 | GLY241 | 8.58 |
| GLY341 | 0.31 | GLY242 | 1.50 |
| GLY348 | 2.85 | GLY277 | 4.24 |
| GLY396 | -0.85 | GLY294 | 2.35 |
| GLY408 | 2.38 | GLY339 | 2.63 |
| GLY411 | 1.24 | GLY345 | 1.74 |
| GLY428 | 2.39 | GLY389 | -0.64 |
| GLY433 | 4.80 | GLY399 | -1.09 |
| GLY443 | 3.0 | GLY411 | 2.74 |
| GLY449 | 3.05 | GLY414 | -0.41 |
| GLY456 | 5.10 | GLY427 | 3.24 |
| GLY479 | 6.03 | GLY431 | 2.06 |
| GLY480 | 4.91 | GLY436 | 4.94 |
| GLY487 | 2.06 | GLY446 | 2.95 |
| GLY491 | 4.78 | GLY452 | 5.75 |
| GLY532 | 1.89 | GLY459 | 6.52 |
| GLY553 | 3.03 | GLY467 | 3.56 |
| GLY554 | 0.35 | GLY470 | -0.60 |
| GLY568 | 0.07 | GLY488 | 2.18 |
| GLY579 | 0.14 | GLY490 | 3.67 |
| GLY605 | -0.22 | GLY491 | 6.61 |
| GLY606 | 3.14 | GLY498 | 5.47 |
|  |  | GLY544 | 2.05 |
|  |  | GLY549 | 1.02 |
|  |  | GLY589 | 2.21 |
|  |  | GLY590 | -0.72 |
|  |  | GLY614 | 3.65 |
|  |  | GLY637 | 1.55 |
|  |  | GLY646 | 3.98 |
|  |  | GLU647 | 0.45 |
