# Supplementary material for: Insight into the Structure, Dynamics and the Unfolding Property of Amylosucrases: Implications of Rational Engineering on Thermostability
Source: PLoS One. 2012 Jul 6;7(7):e40441. doi: 10.1371/journal.pone.0040441 (PMC3391273; doi:10.1371/journal.pone.0040441)
Supplement: Table S2 — The Ala-scan for proline residues of NpAS and DgAS. (DOC) [file pone.0040441.s004.doc]

Table S2 The Ala-scan for proline residues of NpAS and DgAS

| NpAS | | DgAS | |
| --- | --- | --- | --- |
| Residue | ΔΔG (kcal·mol^-1^) | Residue | ΔΔG (kcal·mol^-1^) |
| PRO2 | 1.72 | PRO54 | 2.75 |
| PRO17 | 1.77 | PRO69 | 2.46 |
| PRO41 | 1.97 | PRO83 | 1.48 |
| PRO59 | 2.22 | PRO89 | 2.46 |
| PRO90 | 1.49 | PRO127 | 3.00 |
| PRO120 | 1.77 | PRO131 | 1.59 |
| PRO134 | 2.86 | PRO150 | 1.80 |
| PRO139 | 1.88 | PRO196 | 1.51 |
| PRO157 | 1.91 | PRO206 | 0.48 |
| PRO203 | 1.18 | PRO211 | 2.80 |
| PRO213 | 0.81 | PRO219 | 1.21 |
| PRO218 | 2.86 | PRO223 | 2.30 |
| PRO230 | 2.58 | PRO227 | 0.79 |
| PRO234 | 0.78 | PRO260 | 2.31 |
| PRO262 | 2.80 | PRO301 | 1.78 |
| PRO303 | 1.66 | PRO319 | 2.79 |
| PRO321 | 3.30 | PRO331 | 1.49 |
| PRO333 | 3.08 | PRO380 | 2.25 |
| PRO351 | 2.82 | PRO381 | 0.86 |
| PRO380 | 2.05 | PRO383 | 2.65 |
| PRO435 | 1.82 | PRO430 | 1.33 |
| PRO440 | 1.97 | PRO443 | 1.70 |
| PRO462 | 1.03 | PRO469 | 1.63 |
| PRO482 | 2.47 | PRO493 | 2.36 |
| PRO514 | 1.78 | PRO513 | 1.23 |
| PRO527 | 1.15 | PRO517 | 0.81 |
| PRO549 | 1.25 | PRO525 | 1.76 |
| PRO585 | 1.78 | PRO539 | 1.09 |
| PRO597 | 1.20 | PRO542 | 1.21 |
| PRO619 | 2.49 | PRO561 | 2.03 |
|  |  | PRO573 | 1.80 |
|  |  | PRO575 | 2.28 |
|  |  | PRO587 | 1.40 |
|  |  | PRO605 | 2.68 |
|  |  | PRO631 | 1.37 |
|  |  | PRO649 | 0.68 |
