# Supplementary material for: Insight into the Structure, Dynamics and the Unfolding Property of Amylosucrases: Implications of Rational Engineering on Thermostability
Source: PLoS One. 2012 Jul 6;7(7):e40441. doi: 10.1371/journal.pone.0040441 (PMC3391273; doi:10.1371/journal.pone.0040441)
Supplement: Table S3 — The free energy calculation for the proline residues of NpAS and DgAS. (DOC) [file pone.0040441.s005.doc]

Table S3 The free energy calculation for the proline residues of NpAS and DgAS

|  | Proline residues | | |  | Proline residues | | |
| --- | --- | --- | --- | --- | --- | --- | --- |
| Domain | NpAS | DgAS | ΔΔG(kcal·mol^-1^) | Domain | NpAS | DgAS | ΔΔG(kcal·mol^-1^) |
| N | P2 | - | - | A | P90 | P83 | - |
|  | P17 | - | - |  | P120 | D113 | -0.89 |
|  | P41 | E36 | -1.08 |  | P134 | P127 | - |
|  | P59 | P54 | - |  | C138 | P131 | 1.67 |
|  | N76 | P69 | -1.96 |  | P139 | R132 | 4.00 |
| B | P203 | P196 | - |  | P157 | P150 | - |
|  | P213 | P206 | - |  | P262 | P260 | - |
|  | P218 | P211 | - |  | P303 | P301 | - |
|  | R226 | P219 | -0.5 |  | P321 | P319 | - |
|  | P230 | P223 | - |  | P333 | P331 | - |
|  | P234 | P227 | - |  | P351 | N354 | 0.41 |
| B' | D427 | P430 | -1.0 |  | H377 | P380 | 2.24 |
|  | P435 | V438 | -0.49 |  | N378 | P381 | -1.37 |
|  | P440 | P443 | - |  | P380 | P383 | - |
| C | N560 | P573 | -0.58 |  | P462 | P469 | - |
|  | N562 | P575 | 5.53 |  | P482 | P493 | - |
|  | - | P587 | - |  | S502 | P513 | -1.87 |
|  | P585 | T601 | -0.34 |  | D506 | P517 | 0.77 |
|  | T589 | P605 | -2.11 |  | P514 | P525 | - |
|  | P597 | L613 | 6.9 |  | P527 | P539 | - |
|  | D614 | P631 | -0.69 |  | A530 | P542 | -1.51 |
|  | P619 | G637 | -1.25 |  | P549 | P561 | - |
|  | - | P649 | - |  |  |  |  |
